# Application of double plate fixation combined with Masquelet technique for large segmental bone defects of distal tibia: a retrospective study and literature review

**DOI:** 10.1186/s12893-024-02396-1

**Published:** 2024-04-10

**Authors:** Zhaohui Wang, Chengyou Zou, Xiaohuan Zhan, Xianhui Li, Guocai Ghen, Junqing Gao

**Affiliations:** 1 Affiliated Foshan Hospital of Traditional Chinese Medicine, Guangzhou University of Chinese Medicine, Foshan, China; 2 The Eighth Affiliated Hospital of Chinese Medicine, Guangzhou University of Chinese Medicine, Guangzhou, China

**Keywords:** Masquelet technique, Double plate fixation, Cement spacer, Induced membrane, Distal tibia

## Abstract

**Background:**

There is no effective consensus on the choice of internal fixation method for the Masquelet technique in the treatment of large segmental bone defects of the distal tibia. Thus, the study aimed to investigate the outcomes of the Masquelet technique combined with double plate fixation in the treatment of large segmental bone defects.

**Methods:**

This was a retrospective study involving 21 patients with large segmental bone defects of the distal tibia who were treated between June 2017 and June 2020. The length of bone defect ranged from 6.0 cm to 11 cm (mean, 8.19 cm). In the first stage of treatment, following complete debridement, a cement spacer was placed to induce membrane formation. In the second stage, double plate fixation and autologous cancellous bone grafting were employed for bone reconstruction. Each patient’s full weight-bearing time, bone healing time, and Iowa ankle score were recorded, and the occurrence of any complications was noted.

**Results:**

All patients were followed up for 16 to 26 months (mean, 19.48 months). The group mean full weight-bearing time and bone healing time after bone grafting were 2.41 (± 0.37) months and 6.29 (± 0.66) months, respectively. During the treatment, one patient had a wound infection on the medial side of the leg, so the medial plate was removed. The wound completely healed after debridement without any recurrence. After extraction of iliac bone for grafting, one patient had a severe iliac bone defect, which was managed by filling the gap with a cement spacer. Most patients reported mild pain in the left bone extraction area after surgery. The postoperative Iowa ankle score range was 84–94 (*P* < 0.05). In this cohort, 15 cases were rated as “excellent”, and 6 cases as “good” on the Iowa ankle scoring system.

**Conclusion:**

The Masquelet technique combined with double plate fixation is a safe and effective method for the treatment of large segmental bone defects of the distal tibia.

## Introduction

With increasing incidences of motor vehicle accidents and industrial work-associated cases as well as postoperative infection-related loss of large segments of the tibial bone have been posing a significant challenge to orthopaedic surgeons [1]. Currently, such large bone defects are defined as bone defects of at least 6 cm lesion length. The most commonly used treatment options for large segmental bone defects include distraction osteogenesis, free vascularized fibular grafting (FVFG), and the Masquelet technique [2–5]. Distraction osteogenesis technology refers to the sequential application of bone transport technology and limb re-lengthening process and is considered the “gold standard” for the treatment of large segmental bone defects. Given a relatively longer treatment period and the inconvenience of wearing external fixation supports, a high degree of compliance is required from the patient. Moreover, infections at the pin site and skin opening areas and nonhealing of bone defects have been reported in patients with tibial bone injuries [3, 4]. The FVFG approach requires extreme carefulness in bone dissection and separation of the injured bone and vascular pedicle at the donor site to prevent secondary infections and advanced skills in bone microsurgeries. Hence, this procedure is challenging and time-consuming, requiring a long period for ossification of the fibula and tibia. Besides, there is a risk of stress fracture at the recipient site, so it is difficult to popularize in clinics [4]. In 2000, Masquelet et al. [6] proposed a method of cancellous bone implantation in an induced membrane to treat large tibial segmental bone defects. Compared with other bone reconstruction methods, the FVFG technique offers the advantage of a simple surgical procedure and relatively faster bone reconstruction [7].

The induced-membrane Masquelet technique requires soft tissues under good conditions, as well as robust internal fixation. Presently, intramedullary nailing is recommended in the second stage of this procedure to enhance early weight-bearing and reduce the amount of bone grafting [8, 9]. However, for large segmental bone fractures of the distal tibia, intramedullary nail fixation is difficult and yields poor stability. Currently, there is no effective consensus on the choice of internal fixation methods for the Masquelet technique. Therefore, we adopted a unique approach of using double plates for bone reconstruction and fixation in the second stage, following external fixator-assisted fixation and induced membrane formation in the first stage. In this study, we analyzed the outcomes of the Masquelet technique combined with double plate fixation in the treatment of large segmental bone defects of the distal tibia.

## Methods

### Patient characteristics

Between June 2017 and June 2020, 21 patients with large segmental bone defects of the distal tibia were treated using the Masquelet technique combined with double plate fixation in a single clinic (Table 1). The patient inclusive criteria were as follows: (1) over 18 and under 60 years of age; (2) the range of bone defects in the distal tibia should be at least 6 cm; (3) Masquelet technique with double plate fixation was used, and (4) the patient underwent at least one year of follow-up examinations. The exclusion criteria included patients with concomitant diseases causing (1) multiple fractures; (2) serious nerve and vascular injuries, and (3) severe systemic osteoporosis.

**Table 1:** Patient characteristics

Patient characteristics (*n* = 21)	
Age (Years)	44.19 ± 7.88(range,30–57)
Sex (Male/Female)	14/7
Side(cases) (Left/Right)	10/11
Cause of injury (Traumatic/Infectious)	12/9
Length of defect (cm)	8.19 ± 1.41(range,6–11)
Wound repair	
No	11
Pedicled skin flap	5
Skin grafting	4
Free anterolateral thigh flap	1
Bacterial infection	
negative	12
Pseudomonas aeruginosa	4
Staphylococcus aureus	4
Acinetobacter baumannii	1
latelet-rich plasma (Yes/No)	9/12
Iliac bone extraction area (Left/Right/Bilateral)	4/4/13
Full weight-bearing time (months)	2.41 ± 0.37(range,2–3)
Bone healing time (months)	6.29 ± 0.66(range,5.5–7.5)
Follow-up time (months)	19.48 ± 2.58(range,16–26)
Complications, if any(Yes/No)	2/19

The patients underwent 2 to 5 surgeries for internal fixation, external fixation, debridement, and vacuum sealing drainage before the Masquelet procedure. The study was approved by the institutional review board, and informed consent was obtained from each patient.

### Surgical technique

The treatment was divided into two stages. In the first stage, a thorough cleaning of the lesion area was carried out. Pre-operative computed tomography (CT) and magnetic resonance imaging (MRI) examinations were employed to determine the length of bone defects and bone infection so that an extensive debridement could be performed on necrotic soft tissues and fibrotic scar tissues around the fracture area. Usually, debridement should be applied to 2 mm of normal soft tissue to completely expose the bone lesion site [10]. Bone biting forceps were used to remove sclerotic bones, pus, and inflammatory granuloma until the "chili sign" of fresh blood exudation was visible at the bone fracture end [11, 12]. Following thorough debridement, two 5.0-mm half-pins were placed in the middle and upper tibia, one 5.0-mm half-pin was placed in each of the talus and calcaneus, and the limb was temporarily fixed with an external ankle fixator. After measuring the length, width, and depth of the bone defect area, 40 g of poly(methyl methacrylate) bone cement spacer (Heraeus Medical GmbH, Hanau, Germany) was mixed with vancomycin (1–2 g) to fill the bone defect area, and the medullary cavity, followed by wrapping the edge of the tibial fracture. Since the cement spacer polymerization causes localized heating, the area was repeatedly washed with cold saline to avoid thermal damage to local tissue. Then, the wound was sutured with minimum tension as appropriate, and a suitable flap or skin graft was used to repair the skin defect. On postoperative Day 1, straight leg elevation, isometric contraction of the quadriceps femoris, knee flexion and extension, and other exercises were implemented. In the first stage, weight-bearing was forbidden. Routine postoperative blood tests, including the level of C-reactive protein (CRP), and erythrocyte sedimentation rate (ESR) were conducted regularly. Intravenous antibiotic doses were continued until the infection indicators, such as white blood cell (WBC) count, serum CRP level, and ESR, were reduced to normal levels.

**Fig. 1:**
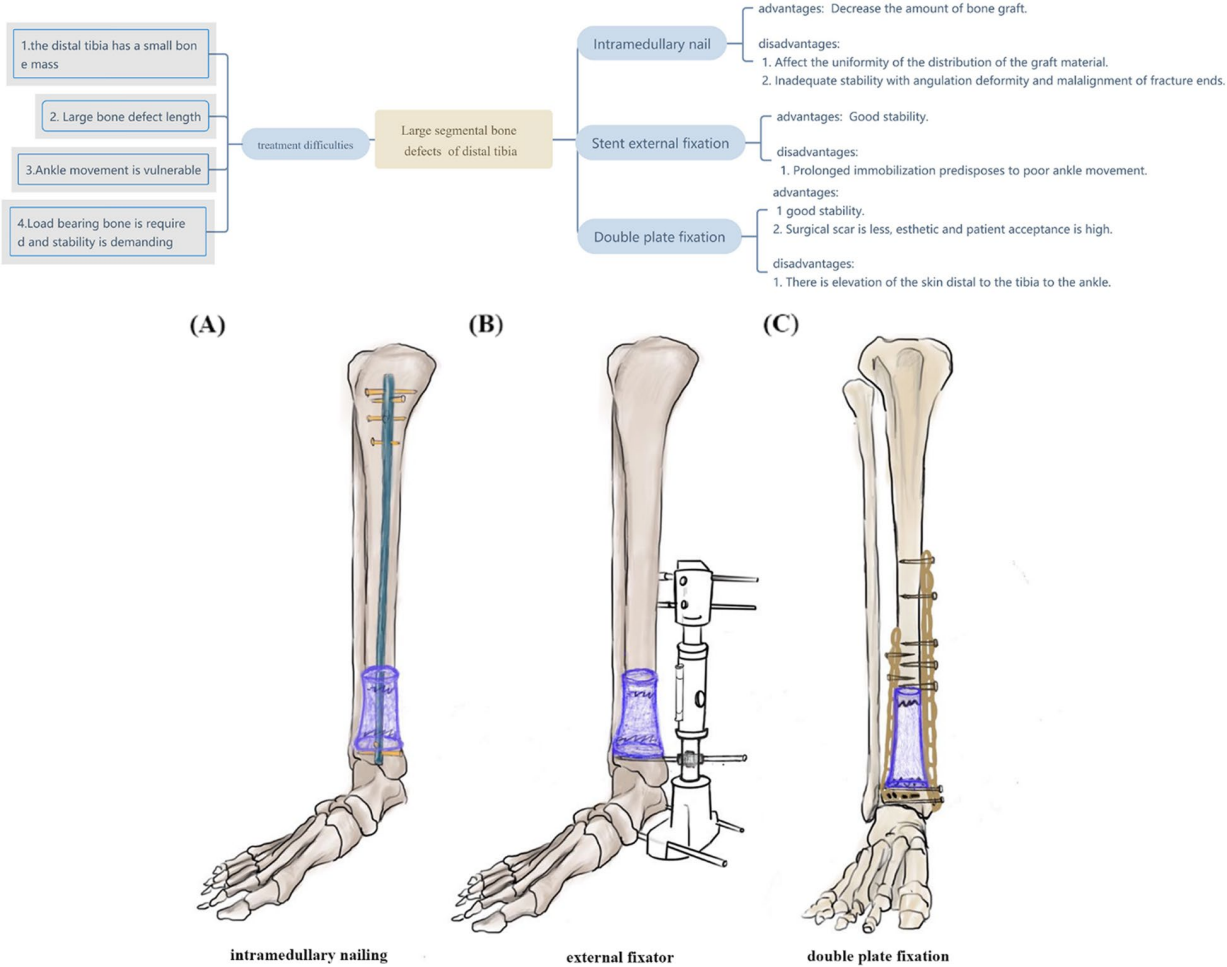
**A** figure simulating the situation of intramedullary nail fixation in a large segmental bone defect of the distal tibia; **B** figure mimics the situation when using external fixation scaffold to fix a large segmental bone defect of distal tibia by ultra articular fixation membrane induction technique in phase II; **C** figure simulating the second stage of large segmental bone defect in the distal tibia treated with double plate fixation and membrane induction technique

At the postoperative 6–8-week, if the soft tissue condition of the affected limb was suitable for internal fixation, and there was no symptom of infection, the second-stage surgery was performed. The skin was cut along the original incision, and the induced membrane was cut longitudinally. The cement spacer was cracked into several pieces using a bone osteotome or bone chisel and was removed with extreme care to prevent damage to the induced membrane. The medullary cavity was drilled with a 2.8-mm drill, decorticated with a thin bone osteotome along the edge of the broken end of the excised bone, and washed repeatedly with normal saline. The length and the force line of the limb were adjusted and fixed with double plates placed on the anterolateral and medial sides of the distal tibia (Fig. 1C). Next, under anesthesia, autologous bone harvest was conducted through an oblique incision of about 2 cm at the posterior edge of the anterior superior iliac spine. Notably, the incision was made close to the bone surface to reduce the damage to peripheral microvasculature and nerves. After stripping the attached soft tissue, the medial plate of the iliac bone mass was removed by cutting in the anterior and posterior sides, followed by filling with cancellous bone using a bone scraper. The drainage was then placed back, and the surgical opening was closed. For bone removal, at least 3 cm of iliac bone was preserved upstream of the anterior superior iliac spine to avoid damage to the joint muscle and inguinal ligament as well as reduce the risk of stress fractures. After removing any residual soft tissues attached to the bone flap, the bone block was broken into granules and stuffed in fractured or defective areas. Pre-operative CT and X-ray images were used to evaluate the autologous iliac bone on the same side. If the bone defect was serious, artificial bone was mixed with allograft bone. The ratio of allograft bone to autologous bone was maintained at less than 1:3. The cancellous bone was extracted from the patient's iliac bone after evaluating the amount of bone graft needed according to the experience of the operator. The cancellous tissue of the iliac bone was crushed into granules and evenly implanted into the bone defect in most cases, however, these granules were mixed with platelet-rich plasma (PRP) in five cases. Then, the induced membrane was sutured as tightly as possible. In the event of a defective induced membrane, the bone graft material was sealed and wrapped with the surrounding muscle membrane or fascia. A drainage tube was retained for postoperative 2 to 3 days.

### Postoperative treatments

After completion of the Masquelet surgery, depending on the wound conditions, broad-spectrum antibiotics were administered for one week. The affected limb was fixed in the functional position with plaster for 4 weeks, and a simple splint was used for the following 4 weeks. The repair was examined regularly using X-rays, and the appropriate period of weight-bearing was determined according to the pain in the extraction area of the iliac bone and the bone reconstruction in the bone graft area.

### Observational indicators

Bone healing was defined as the efficient union of bone fractures [13], X-rays revealing a continuum of at least three of the four cortices of minimum 2 mm thickness and absence of pain in the distal tibia. During the final follow-up session, patients completed the Iowa ankle questionnaire [14], where a score of 90–100 represented an excellent, 80–99 a good, 70–79 a fair, and less than 69 as poor results.

### Statistical analysis

Statistical analysis of all collected data was performed using SPSS software (version 26.0; SPSS; Chicago, IL, USA). Data were statistically described as mean ± standard deviation (SD) or percentage (%). The pre-operative and postoperative Iowa scores were compared using a paired sample t-test. A *P*-value of less than 0.05 indicated a significant level of difference.

## Results

**Fig. 2:**
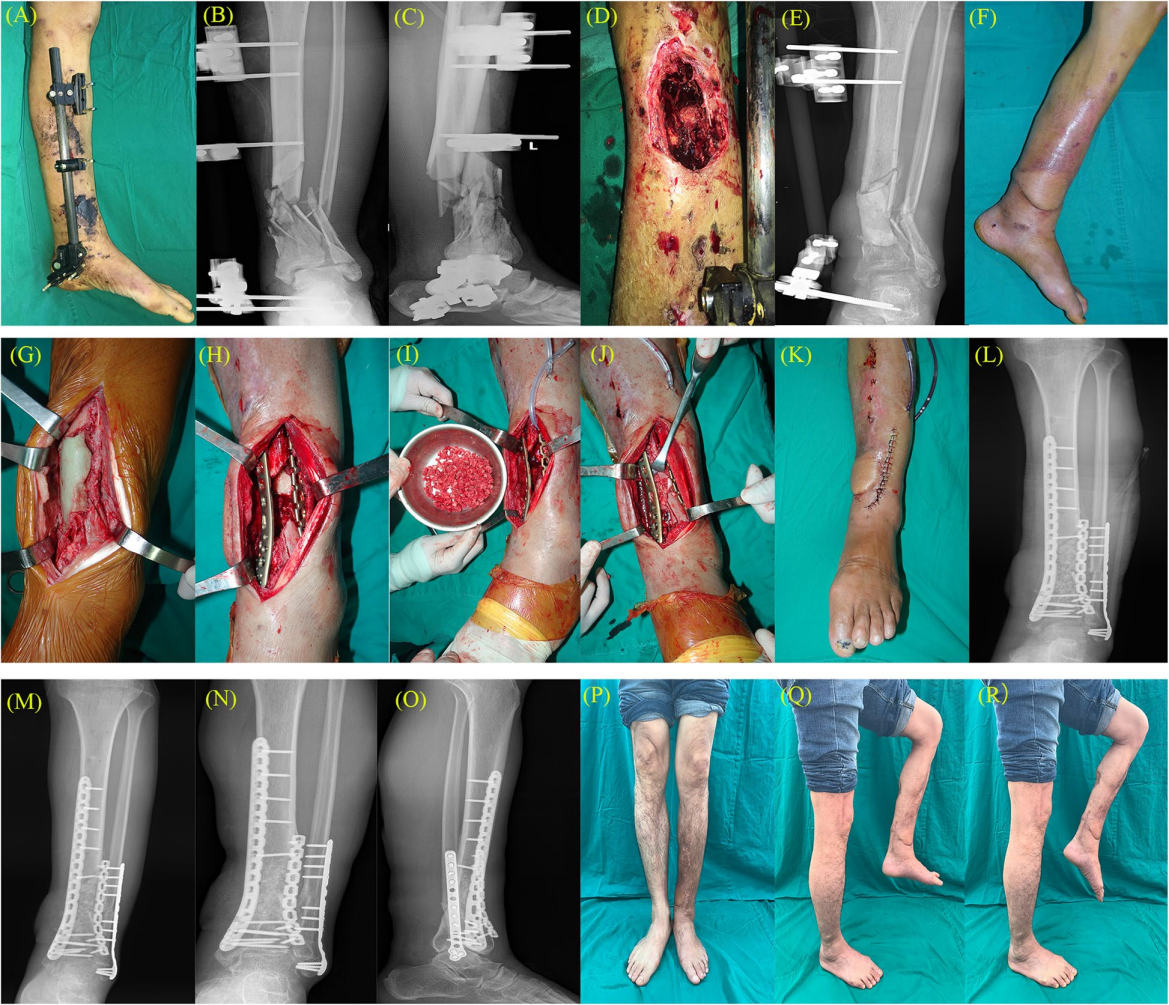
**A**/**B**/**C**/**D** The patient was transferred from an external hospital after being injured. The left medial leg was infected, the necrotic soft tissue and infected bone were cleared, and Staphylococcus aureus was detected. **E**/**F** The ipsilateral peroneal artery perforator flap was used to reconstruct soft tissue defect. **G**/**H**/**I**/**J**/**K** The induced membrane was cut longitudinally and the cement spacer was removed. The distal tibia was fixed with double plates, and iliac bone was removed to make bone granules, which were packed into the defective tibial region. **L** Postoperative X-ray after bone grafting. **M** Follow up X-ray one year after bone grafting surgery. **N**/**O** Follow up X-ray after bone grafting for 2 years. **P**/**Q**/**R** After 26 months of surgery, the patient's left calf recovered well.The Iowa ankle score was 86

Our cohort included 14 male and 7 female patients with the distal tibia injury. The mean age of patients was 44.19 years (range, 30–57 years). There were 12 cases of traumatic fractures and bone defects caused by the high-energy injury and another nine cases of bone defects caused by infection at the fracture site after internal fixation. 11 patients had right-side defects, whereas the rest had left-side defects. 10 patients required orthoplastic soft tissue reconstruction – five cases with a pedicled skin flap, one with a free anterolateral thigh flap, and four with a free skin graft (Fig. 2). Bacterial infection was detected in 9 patients: one case of *Acinetobacter baumannii*, four cases of *Staphylococcus aureus*, and four cases of *Pseudomonas aeruginosa*. The range of tibial bone defects was 6.0–11 cm (mean, 8.19 cm). All patients were followed up for 16–26 months (mean, 19.48 months). The group mean full weight-bearing time and bone healing time after bone grafting were 2.41 (± 0.37) and 6.29 (± 0.66) months, respectively (Table 1).

**Fig. 3:**
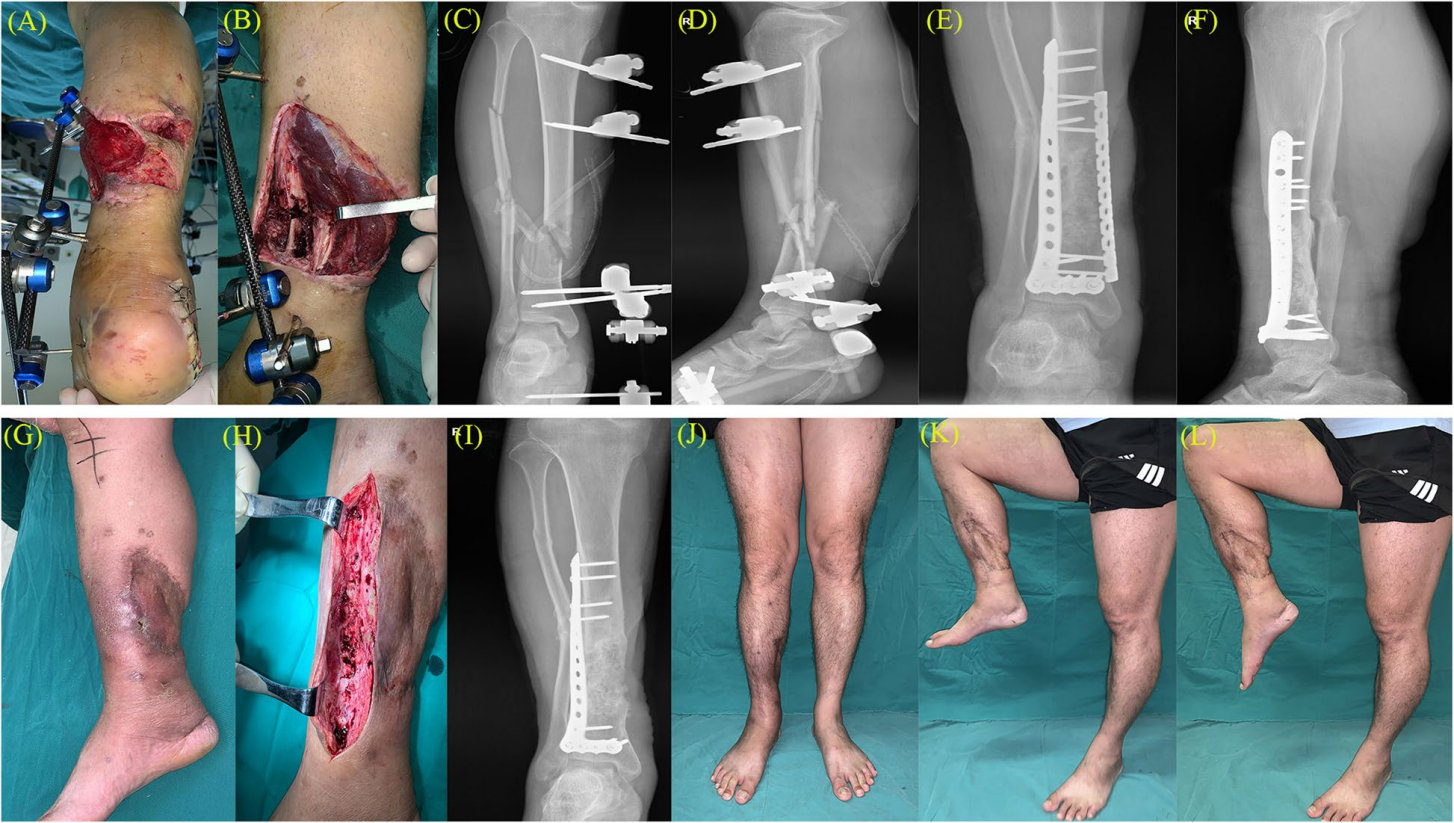
**A**/**B**/**C**/**D** Appearance of the affected limb and X-ray of the right calf when the patient is transferred from another hospital. **E**/**F** X-rays showed that the bone healed well at 13 months after bone grafting. **G**/**H** Redness, swelling and a small amount of exudation were found on the inner side of the right leg, and the plate on the inner side of the right leg was removed. **I** X-rays showed that no infection in the tibia at 3 months after removing the medial plate. **J**/**K**/**L** The Iowa ankle score was 86 at 6 months after removing the medial plate

**Fig. 4:**
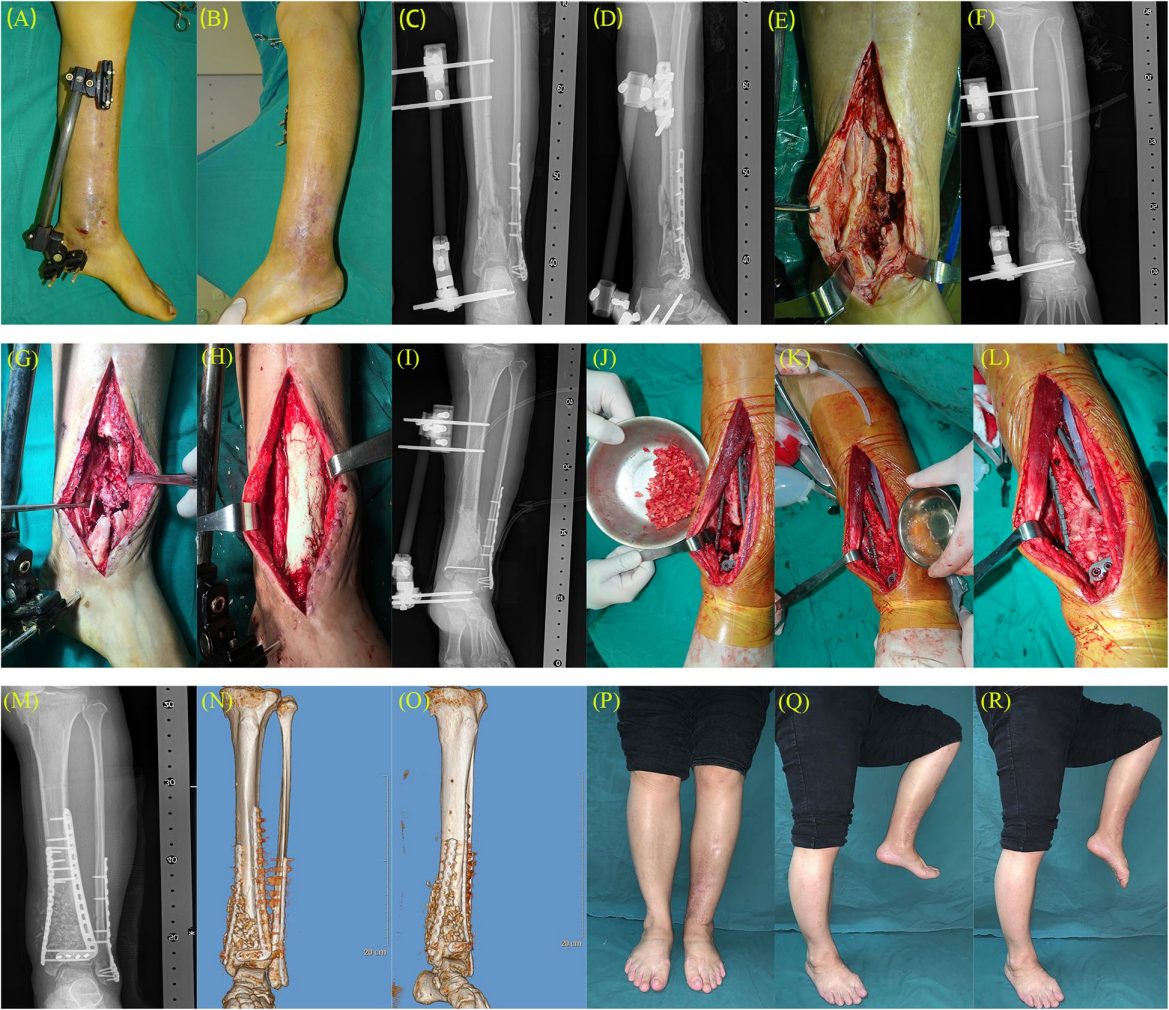
A 44-year-old woman’s left leg was injured by a heavy object, resulting in fracture of the tibia and fibula and fluid exudation for a period of over 3 months. **A**/**B**/**C**/**D** Appearance and X-ray of the patient's left calf upon admission. **E**/**F** Left calf expansion wound and postoperative X-ray. **G**/**H**/**I** One week later, the tibial bone defect was filled using cement spacer. **J**/**K**/**L** Six weeks later, the induced membrane was cut longitudinally and the cement spacer was removed. The distal tibia was fixed with double plates, and iliac bone was removed to make bone granules, which were mixed with PRP and packed into the defective tibial region(K Using PRP). **M**/**N**/**O** X-ray and CT reconstruction show bone reconstruction after bone grafting. **P**/**Q**/**R** 21 months after bone grafting, the foot and ankle function recovered well.The Iowa ankle score was 94

During the treatment, one patient had a wound infection on the medial side of the leg, so the medial plate was removed. The wound healed after debridement, and the infection did not recur (Fig. 3). After extracting the iliac bone tissue for grafting, one patient had extensive iliac bone defects, which were immediately filled with cement spacer. Distal tibial bone defects were finally repaired in all patients, resulting in a satisfactory outcome. The Iowa ankle scores ranged from 84 to 94. Postoperatively, 15 patients scored “excellent” and six were “good” on the Iowa scale (Table 2). Typical cases are shown in Fig. 4.

**Table 2:** Iowa ankle score and evaluation (*n* = 21)

	Pre-operation	The last follow-up	T /X^2^ value	*P* value
Iowa ankle score	39.52 ± 3.03	90.95 ± 2.94	-117.01	< 0.001
Iowa ankle evaluation				
Excellent	0	15(71.4%)	21	< 0.001
Good	0	6(28.6%)		
Fair	0	0		
Poor	21(100%)	0

## Discussion

Distal tibial bone defects may be caused by high-energy traumatic injuries, bone infections, or other etiological factors related to the tibia’s location and structure. Segmental bone defects are prevalent in 68–79% of distal tibial fracture cases [15]. The major downsides of treating such patients are complex surgical procedures, relatively longer recovery periods, risks of secondary stress fractures at the defect site, internal microbial infections at the fixation sites, and mental trauma. Masquelet et al. [6] have reported successful bone repair using a novel method of bone reconstruction. The method involves the induced formation of periosteum-like material by filling the bone defect area with poly(methyl methacrylate) in the first stage, followed by a thorough debridement and implantation of cancellous bone into the induced membrane. The unique biological and structural characteristics of the induced membrane render the length for bone reconstruction independent of the bone growth rate [16]. Compared with autogenous bone grafting, distraction osteogenesis, and FVFG, the Masquelet technique is less complicated and involves fewer side effects and a relatively reduced infection rate.

The Masquelet technique is divided into two stages. In the first stage, infected bone is completely removed, the limb length and bone defect space are maintained, and the defective area of bone is filled with cement spacer to induce membrane formation. After 6–8 weeks of cement spacer tamponage, a membrane formation is induced, facilitating the supply of blood, nutrients, and osteogenic factors that support effective healing of the bone graft [17, 18]. Osteogenesis occurs in the second stage, which involves the removal of the cement spacer and implantation of autogenous cancellous bone particles into the defective area of the bone, combined with effective internal and external fixation.

Cement spacer tamponage is the key to the formation of an induced membrane. For infected patients, we routinely mix cement with vancomycin in a 40:1 ratio in the first stage, while for uninfected patients, cement alone was used to fill the bone defect. In this study, molding was achieved by filling the cement spacer in the medullary cavity and extending beyond the edge of the fractured end to expand the contact area between the cement spacer and healthy bone. This process provides robust stability of the fixation, owing to proximal and distal intra- and extramedullary attachments. The micromotion that occurs when the interface between the cement spacer and natural bone is not aligned correctly leads to the formation of a fragile and poorly vascularized membrane [19]. Because of the heat released by cement spacer polymerization, a large volume of normal saline can be needed to wash the area repeatedly and avoid heating-induced damage to the molding process [20]. For suspected cases of infection, even if the first-stage operation is successful, the recommended approach is to replace the cement spacer within 4 weeks, until the wound is completely healed and the serum CRP level is restored to the normal level [21]. If the infection relapses, debridement of the membrane and surrounding soft tissue is recommended before repeating the technique [22].

Maintenance of the limb stability is an important part of this treatment outcome. Therefore, we used external fixation to maintain limb stability in the first stage. This method offers the advantages of a simple procedure and a wide range of applications but also has the disadvantage of being uncomfortable for the patient. Sufficient stability is essential for maintaining reduction and promoting the healing and vascular reconstruction of the submembranous graft [22]. Gouron et al. [23] have reported bone nonunion in 35% of 14 children treated using the Masquelet technique and attributed this finding to the lack of either appropriate external or internal fixation or to incorrect cement spacer treatment at the end of the bone. In the second stage, intramedullary nailing is the most commonly used approach for stabilization, following the use of an external fixator and plate [21]. For patients with distal tibial bone defects of greater than 6 cm and requiring preservation of the ankle joint function, we recommend using double steel plates for internal fixation in the second stage, compared to external fixation brackets and intramedullary nail fixation methods. We recommend first analyzing if a patient with a bone defect of greater than 6 cm in the distal tibia and requiring preservation of ankle joint function can be treated with intramedullary nail technology (Fig. 1), or if insufficient bone mass cannot support the implementation of intramedullary nail technology during distal locking. If intramedullary nail technology is used, there can also be a great amount of instability due to low bone mass in the distal end. Therefore, we did not conduct a control experiment comparing the intramedullary nail technique with the double steel plate fixation method. If a super joint stent is used for fixation in the second stage, and the patient complains of ankle stiffness, it goes against the patient's requirement to preserve ankle function and can greatly affect the patient's quality of life. However, if double steel plates are used for fixation, the stability can be much better than that of a single steel plate, thus maximizing the chances of preservation of the patient's ankle joint function. Therefore, we believe that for patients with longer (> 6 cm) distal tibial bone defects and requiring preservation of ankle joint function, it is more appropriate to employ a double steel plate internal fixation procedure in the second stage of membrane induction.

The Masquelet technique requires good skin condition around the area of defective bones. Dugan et al. [24] emphasize that bone reconstruction can only be performed after any soft tissue injury has completely healed. For patients with large bone defects in the distal tibia who use double steel plate internal fixation, there are certain requirements for the soft tissues of the distal tibia. According to our experiences, the soft tissue condition should be assessed first. For patients without any soft tissue damage, judgment should be made based on the skin integrity (intact or broken), softness (such that the thumb and index finger could pinch the anterior tibial skin about 0.5–1 cm), and the ductility to meet the second-stage suture. Even in some patients with slightly insufficient skin ductility, we can appropriately place bone cement in the bone defect areas, increasing about 10–20% on the original basis, forming a cylindrical sleeve, and wrapping both ends of the broken bone. The principle of skin tension can be used to make a preset for the placement of double steel plates. For patients with soft tissue defects, the design of skin flap transplantation closure should be appropriately greater than the actual soft tissue defect area. If the flap survives well, the wound is expected to be completely healed without any swelling or pain symptoms. These criteria represent that the affected limb soft tissue conditions meet the requirements of the second phase of surgery. Of the 21 patients in this cohort, 10 patients required orthoplastic soft tissue reconstruction.

We applied the double plate fixation method, allowing firm fixation and effectively avoiding stress-induced secondary fractures, which can affect the overall bone graft healing. Importantly, excessive fixation can also lead to significant stress shielding, ultimately affecting graft fusion. During treatment, one patient had a wound infection on the medial side of the leg. The patient recovered after removing the medial plate and a dressing change (Fig. 3). For patients with large segmental bone defects in the distal tibia, and are treated with membrane induction technology combined with double steel plate fixation, whether it is necessary to remove a portion of the steel plate after a period of bone reconstruction and if yes, then at what time point, will be our future research direction.

Autologous cancellous bone granule transplantation is a key step in bone reconstruction. Direct complications may result from iliac bone extraction, and there are several pitfalls of the induced membrane technique [25]. For one of our patients, we removed a large amount of bone from the ilium and used a cement spacer to fill the iliac bone during the surgery. In this case, the bone graft volume completely covered the area of defective bone, and an appropriate quantity of cancellous bone particles was packed in the bone fracture end. Therefore, the volume of the bone graft should be sufficiently large to cover the whole area of the defect, however, its size should not prevent membrane closure and revascularization of the graft [26, 27]. Recently, bone induction aids, such as bone morphogenetic proteins, PRP, or bone marrow aspirate concentrates, have been used in combination with the Masquelet technique to increase the bone healing rate [28]. The PRP in this study was obtained using a TriCell PRP preparation device and prepared by double centrifugation. The mixture of PRP and cancellous bone particles was conducive to the adhesion of cancellous bone particles. Moreover, PRP is rich in growth factors, which are essential to promoting bone healing [29]. Of the 21 patients in this group, nine patients agreed to use PRP, and there were no adverse reactions related to this treatment.

For patients undergoing the Masquelet technique, the postsurgical period required before weight-bearing remained unclear. Weight-bearing at an early stage can lead to proximal bone loss, while delayed weight-bearing may affect the bone healing efficiency and function. We usually encourage active functional exercise on the first day after the operation and walking with no load for the following two weeks. Owing to the strong fixation of double plates at the distal tibia, our patients were gradually loaded with the protection of a brace for the postoperative 2–3 months.

## Advantages and limitations

Advantages: For patients with large segmental bone defects in the distal tibia, the Masquelet technique can be useful and effective. In the choice of secondary fixation mode, dual plates are not only helpful to stabilize the fixation robustly but also make the surgery procedure easy to operate. Moreover, this process allows a reduction in surgical incision area and increases the patient’s postoperative aesthetics, which are more acceptable to patients.

Limitations: The use of dual plates on the distal tibia can greatly increase the requirement of skin graft area on this segment. Due to the insufficient number of cases and the lack of a longer follow-up period, further clinical studies will continue to be performed exploring the requirements of a successful dual plate technique for skin thickness, viability, etc.

## Conclusion

Satisfactory treatment outcomes were achieved using the Masquelet technique combined with double plate fixation to repair the large segmental bone defects of the distal tibia.

## Data Availability

The data set reported in this study may be obtained from the corresponding author upon reasonable request.
